# A scoping review of public hospitals autonomy in Iran: from budgetary hospitals to corporate hospitals

**DOI:** 10.1186/s12913-021-06620-z

**Published:** 2021-07-06

**Authors:** Rahim Sohrabi, Sogand Tourani, Mehdi Jafari, Hossein Joudaki, Leila Doshmangir, Javad Moghri, Nicola Luigi Bragazzi

**Affiliations:** 1 Department of Health Services Management, School of Health Management and Information Sciences, Iran University of Medical Sciences, Tehran, Iran; 2 Iranian Social Security Organization, Zanjan Province Health Administration, Zanjan, Iran; 3 Department of Health Economics and Planning, Iranian Social Security Organization, Tehran, Iran; 4 Department of Health Policy & Management, Tabriz Health Services Management, School of Management & Medical Informatics, Tabriz University of Medical Sciences, Tabriz, Iran; 5 Social Determinants of Health Research Center, Mashhad University of Medical Sciences, Mashhad, Iran; 6 School of Public Health, Department of Health Sciences (DISSAL), University of Genoa, Genoa, Italy

**Keywords:** Hospital, Organizational reform, Governance, Board of trustees, Health policy, Iran

## Abstract

**Background:**

Organizational reforms of hospitals in Iran are mainly aimed at improving efficiency, reducing government spending on health care, and improving the quality of services. These reforms began with hospital autonomization and have continued with other initiatives such as formation of board of trustees, independent and corporatized hospitals.

**Objective:**

The purpose of this scoping review was to summarize and compare the results of studies conducted on organizational reform of hospitals in Iran to paint a more clear picture of the status quo by identifying knowledge gaps, inform policymakers, and guide future studies and policies.

**Method:**

This review’s methodology was inspired by Arksey and O’Malley’s methodological framework to examine the extent, range, and nature of research activity about organizational hospital reforms in Iran. A literature search was performed using PubMed, Scopus, Web of Science, and Google Scholar for English papers as well as SID, IranDoc, Magiran, and the Social Security Research Institute Database for Persian papers from 1991 to April 2020.

**Results:**

Twenty studies were included in the review. Studies were grouped by the types of organizational reform, study’s objective, setting, methodology, data collection and analysis techniques, and key findings. Thematic construction was used based on the types of organizational reform to present a narrative account of existing literature.

**Conclusions:**

The autonomy granted to the hospitals was unbalanced and paradoxical in terms of key effective dimensions. Poor governance and regulatory arrangements, low commitment to corporate governance, Inappropriate board composition, weak internal controls, unsustainable financing and inefficient payment mechanisms, poor interaction with stakeholders and ignoring contextual factors have been cited as the main reasons for the failure of organizational reforms in Iran. The limited use of evidence and research was obvious at different stages of policymaking, especially in the policy formulation phase and evaluation of its results.

## Introduction

Governments around the world are implementing health reforms, in order to cope with issues like rising costs, user dissatisfaction with services, and problems that are often associated with public hospitals such as technical and allocation inefficiencies, low productivity, non-accountability to patients, waste, fraud, and corruption [1]. While privatization seems to be a good solution to the problems of public hospitals, many countries are reluctant to pursue it for a variety of reasons. First, privatization may lead people to think that the government is not honoring its obligations to provide healthcare to its population. Second, public ownership of hospitals may seem to be a better alternative for achieving objectives other than efficiency and quality improvement. The third reason is the failure of many privatization attempts in low- and middle-income countries [2].

Healthcare and hospital care sectors are different from other industries and should be regulated by the government to ensure universality, equity and accessibility for all, and cost-effectiveness without wasting resources [3].

As a result, many countries are pursuing organizational reforms of public hospitals as an alternative. These reforms are often called autonomization or corporatization and commonly involve maintaining public ownership of hospitals while transforming them into a more independent entity responsible for their performance. In other words, the structures, incentives, and competitiveness of the private sector are applied to public hospitals with the expectation that market pressures will result in better performance outcomes, higher efficiency, and quality [2].

Views about the role of the state in socioeconomic development have changed in recent years, and organizational hospital reforms are a major example of that [4]. In Iran, these reforms have been a function of the domestic and international macroeconomic contexts. Following global trends and using 5-year development plans, the Iranian government has been applying the principles of new public management, including downsizing, managerialism, decentralization, de-bureaucratization, and privatization [5, 6]. The health sector has also undergone these reforms and various policies have been developed and implemented to improve hospital performance. These include widespread managerial, technological, financing (budgeting and payment), and organizational reforms [7].

The Ministry of Health and Medical Education (MOHME) and the Social Security Organization (SSO) as the most important and the largest healthcare providers in Iran have implemented various initiatives aimed at organizational hospital reform. The majority of hospitals in Iran are budgetary or traditional public hospitals. These hospitals are usually run bureaucratically by a chairman and manager. These hospitals are part of the government and the hospital manager is necessarily an “administrator”. They have limited decision-making power over the human, physical, and financial resources and strategic management of the hospital, and are often controlled through government hierarchies, laws, and regulations. The purpose of organizational reform is to give these hospitals some degree of management autonomy. Hospital autonomization, board of trustees (BT) hospitals, and independent hospitals policy in hospitals affiliated with the MOHME and corporatization of hospitals affiliated with the SSO are some of the most important policies that have been implemented in the Iranian health system, characterized by the common themes of decentralization and downsizing of the public sector.

The central question of this research is: What is known from the existing literature about organizational hospital reforms in Iran, and what are the main factors that lead to the failure of reforms? Some various studies and reports have examined different aspects of these policies and the purpose of the present research is to clarify the existing body of research evidence on organizational hospital reforms in Iran. The major findings of the studies retrieved and included are summarized to draw a complete picture of the status quo to inform policymakers and researchers. Finally, knowledge gaps are identified to guide future studies.

## Methods

### Eligibility criteria

This review is based on the methodological approach of Arksey and O’Malley [8] to examine the extent, range, and nature of research activity about hospital organizational reforms in Iran, including the development of autonomous, board of trustee, independent, and corporate hospital policies in Iran. Peer-reviewed papers and the grey literature (government reports, policy documents, reports of consultants, unpublished reports, etc.) between 1991 and April 2020, written in English and Persian, were included. Databases of ongoing research and unpublished literature were also searched.

### Information sources

A literature search was performed using PubMed, Scopus, Web of Science, and Google Scholar for English papers as well as SID, IranDoc, Magiran, and the Social Security Research Institute Database for Persian papers and gray literature. Ongoing research and unpublished literature were also included. A Google search with no date restrictions was also conducted and only the first 200 hits (as sorted by relevance by Google) were screened. The search strategies were drafted by an experienced researcher (LD) and further refined through team discussion. The search strategy for PubMed is presented in Table 1. The final search results were exported into EndNote and duplicates were removed. A keyword search strategy was employed using the terms “organizational reform”, “autonomy”, “corporate”, “board of trustees” and “hospital” in English databases and Persian equivalents of these terms including “ESLAHAT-SAZEMANI”, “KHODGARDAN OR MOSTAGHEL”, “SHERKATI OR HEYAT-MODIRE”, “HEYAT-OMANA” and “BIMARESTAN” were used for searching in Persian databases.

**Table 1:** Search strategy summary for the scoping review

Inclusion criteria	***•*** Publication years 1991–2020 ***•*** Peer-reviewed publications, gray literature, government reports, reports of consultants, policy documents, unpublished reports ***•*** English and Persian language; focused on hospital autonomy in Iran
Exclusion criteria	***•*** Editorials/commentaries, letters, conference abstracts ***•*** Papers focused on privatization or public-private partnerships
PubMed search string (final version)	***•*** Search (((((((((((((((((“organizational reform”) OR autonomy [Abstract]) OR autonomization [Abstract]) OR autonomisation [Abstract]) OR corporate [Abstract]) OR corporatization [Abstract]) OR corporatisation [Abstract]) OR corporation*[Abstract]) OR decentralization [Abstract]) OR decentralisation [Abstract]) OR board of trustees*[Abstract]) OR board of directors*[Abstract]) AND hospital) AND Iran)))))

### Selection of sources of evidence

Search results were exported to EndNote X8 reference manager software (Thomson Reuters, Philadelphia, PA, USA) and duplicates were removed. The title and abstract of the articles were independently reviewed by two authors (RS and LD) to screen for eligibility. Articles meeting the inclusion criteria underwent two full-text independent reviews by two authors (RS and LD). In case of disagreements, a third-party reviewer (MJ) would be consulted. Finally, the reference lists of all of the included studies were checked for additional relevant studies.

### Data charting process

Two team members (RS and LD) involved in the review study protocol development independently extracted data from two studies and met to compare their data entries. The final version of the data extraction form was sent via email to team members and modified as required based on feedback from the team.

Subsequently, each included study was abstracted by one team member (RS), and verified by a second reviewer team (LD, HJ and, JM). As an additional data cleaning step, a third reviewer team (MJ, ST and, NLB) verified all the changes made by the second reviewer to ensure data accuracy. Once all studies had been checked for accuracy in a detailed table, a summary table was created for publication using Microsoft Word 2016. The extracted data included report characteristics (e.g., first author, year of publication, publication type, report design), type of organizational reform policies, as well as key findings and implemented policies. All authors reviewed and edited the manuscript and approved the final draft of the manuscript.

### Critical appraisal of individual sources of evidence

As per guidance on conducting scoping reviews and consistent with scoping reviews on health-related topics, the methodological quality of the included reports was not appraised [9].

### Synthesis of results

The studies were grouped by the types of organizational reform, study objective, setting, methodology, data collection techniques, and key findings. Also thematic construction was used based on types of organizational reform to present a narrative account of existing literature.

### Results

**Fig. 1:**
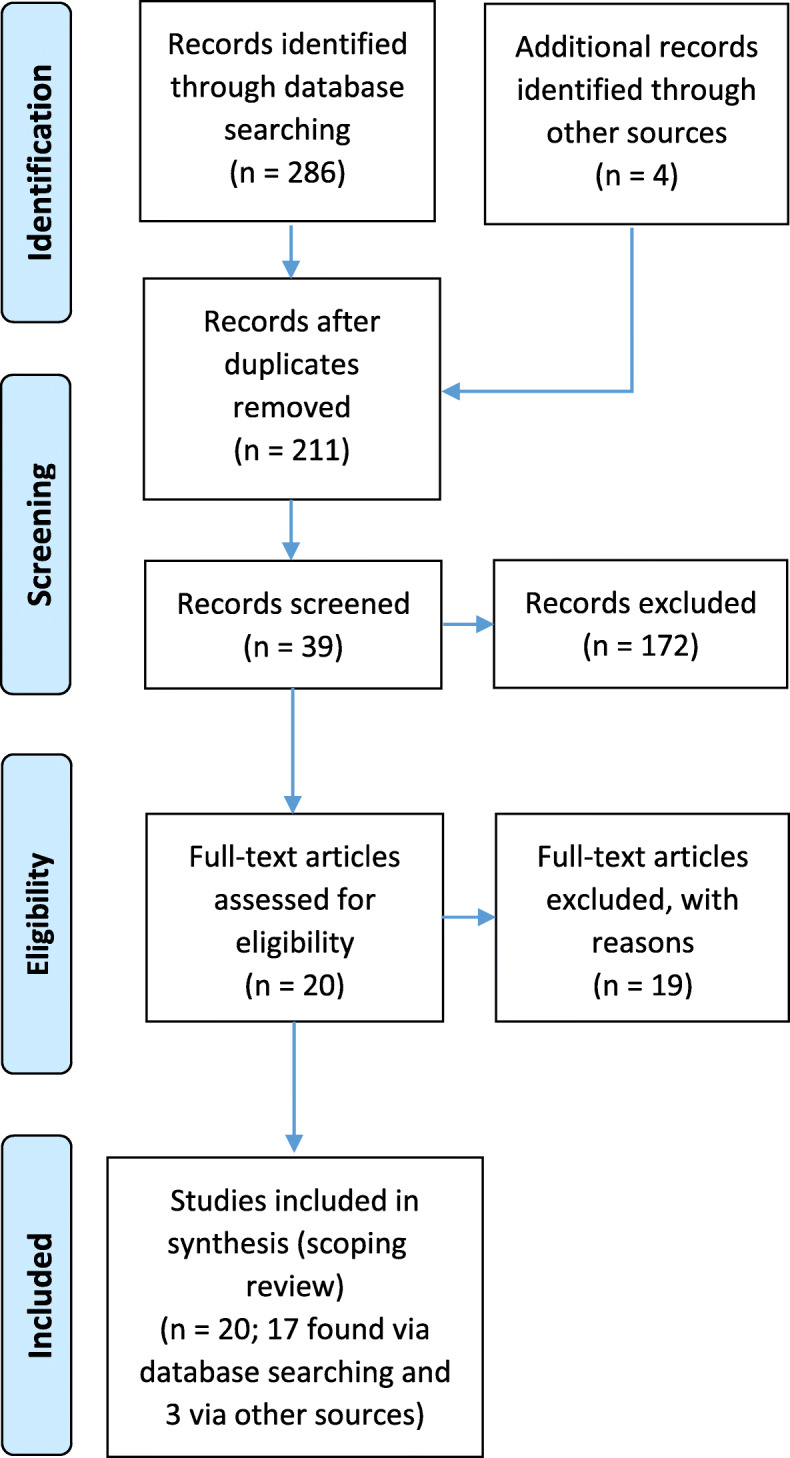
PRISMA flow diagram for included studies

Twenty studies were included in this review. Figure 1 outlines the flow of studies through the inclusion process and Table 2 describes the included studies. Most studies were published articles (16/20; 80%). Most of them were published between 2011 and 2015 (12/20; 60%). There has been only one published study over the period of 10 years after the implementation of the first major policy for organizational hospital reform in Iran, and only two studies 15 years after its implementation. Most studies have focused on BT hospital policy (10/20; 50%), while hospital corporatization policy has been studied the least (3/10; 15%). There are no studies on the new independent hospital policy as its implementation has been ongoing since 2017 and is yet to be fully established. Most studies were conducted in Tehran (4/20; 20%) and Isfahan (4/20; 20%) and most of them were qualitative (10/20; 50%). Most studies were written in English (12/20; 60%), with Mehdi Jafari as the first author of 5 articles (25%) and Leila Doshmangir as the first author of 3 articles (15%), being among the most influential researchers in the area of organizational hospital reforms in Iran.

**Table 2:** Description of included studies

Variables	n (%)
**Publication type**	
Journal article	16 (80)
Thesis	2 (10)
Organizational report	1 (5)
Research project	1 (5)
**Publication date**	
1995–2000	1 (5)
2001–2005	0 (0)
2006–2010	1 (5)
2011–2015	12 (60)
2015–2020	6 (30)
**Policy type**	
Autonomization policy	6 (30)
BT policy	10 (50)
Corporatization policy	3 (15)
Two or more policies	1 (5)
**Setting**	
Tehran	4 (20)
Isfahan	4 (20)
Tabriz	2 (10)
Kerman	1 (5)
Gilan	1 (5)
Two or more provinces	4 (20)
Not applicable	4 (20)
**Methodology**	
Qualitative	10 (50)
Quantitative	7 (35)
Mix-method	3 (15)
**Language**	
English	12 (60)
Persian	8 (40)
**Influential Researchers**	
Mehdi Jafari	5 (25)
Leila Doshmangir	3 (15)
Arash Rashidian	3 (15)
Iravan Masoudi Asl	3 (15)
Amirhossein Takian	3 (15)
Mohammad Arab	3 (15)
Hamid Ravaghi	2 (10)

Table 3 provides the characteristics of published studies on organizational hospital reforms in Iran. In the next section, the results of these studies are examined using Preker and Harding’s conceptual framework and based on the degree of autonomy granted to public hospitals.

**Table 3:** Characteristic of included studies in the scoping review in the order of publication date

First Author, Year	Publication Type, Policy	Study Objective	Setting	Methodology, Data Collection
Amiri 1997 [10]	Thesis, autonomization policy	• Investigating the effect of hospital autonomy on performance indicators before and after the implementation of the policy	5 teaching hospitals (Tehran)	• Quantitative (retrospective before-after study) - Data collection forms (data on performance indicators of 5 teaching hospitals) - Review of 410 medical records to evaluate the rate of readmissions due to surgical site infections
Jafari 2008 [11]	Article, autonomization policy	• Identifying key organizational dimensions that influence the autonomy of university hospitals and the level of autonomy granted in each dimension	6 hospitals (Tehran)	• Qualitative - Semi-structured interview with 27 hospital managers
Jafari 2011 [12]	Article, autonomization policy	• Assessing the views of senior managers in selected hospitals on the degree of autonomy granted to the hospitals	6 hospitals (Tehran)	• Qualitative - 4 initial in-depth interviews and 27 semi-structured interview with hospital managers
Sajadi 2012 [13]	Article, board of trustees policy	• Examining the effect of BT policy on hospital efficiency	1 hospital (Isfahan)	• Mixed method (A quasi-experimental retrospective and qualitative case study) - Performance indicator data were extracted from the hospital’s statistical information resources - Interviews with seven experts in the field (three national policymakers, two local managers, and two hospital administrators)
Manavi 2012 [14]	Article, board of trustees policy	• Estimating the financial impact of implementing the board of trustees policy in Iran	Insurance organizations and the MOHME	• Quantitative (cross-sectional, economic analysis) • Data were extracted by data gathering forms (Insurance organization data, physician’s income, hospital incomes and estimation of total costs)
Gholipour 2013 [15]	Article, board of trustees policy	• Comparing performance indicators of two gynecology hospitals with different organizational governance (budgetary/BT)	2 gynecology hospitals (Tabriz)	• Quantitative, a retrospective longitudinal study • Study variables included: average length of stay (ALOS), bed occupancy rate (BOR), and bed turnover ratio (BTR). The data were extracted via the hospital information systems (HIS) within the hospitals’ admission and discharge units during the period 2010–2012
Ferdosi 2013 [16]	Article, board of trustees policy	• Evaluating the responsiveness of BT hospitals according to World Bank’s organizational reform model (Preker Model) in Isfahan	2 BT hospital (Isfahan)	• Qualitative • Semi-structured interviews with top managers of selected hospitals
Ahmadi 2014 [17]	Article, autonomization policy	• Investigating the effect of autonomization policy on patient satisfaction (as an indicator of service quality)	2 autonomous hospitals (Isfahan)	• Quantitative (cross-sectional study) - Data were collected before and after implementation of the autonomization policy using questionnaires based on SERVQUAL model
Markazi-Moghaddam 2014 [18]	Article, board of trustees policy	• Exploring the obstacles and barriers that lead to the failure of hospital autonomization (BT policy) reform and their mechanisms	All 54-university hospitals that had been granted autonomy in Iran	• Qualitative - Data collection was done within 2 phases: (1) 276 unstructured questionnaires inquiring key informants of barriers, and (2) 23 semi-structured interviews with key informants.
Meshki 2014 [19]	Article, hospital autonomization policy	• Determining the relationship between the autonomy policy and public hospitals’ performance	5 hospitals (Gilan)	• Quantitative (survey study) - 48 questionnaire completed by accounting staff, managers, and physicians.
Doshmangir 2015 [20]	Article, autonomization policy	• Developing a policy map that includes important dates and events leading to the policy process milestones • Understanding intentions and motives of policymakers, general outcomes of the policy, and the reasons behind the perceived failure in achieving the intended objectives • Drawing broader lessons about the viability of the hospital autonomization policy in Iran	Policy-making institutions in the health sector including Medical Council, the Parliament, medical universities, Vice-presidency for Strategic Planning and Supervision, the Cabinet, MOHME, MOSSW, MPs and related agencies	• Qualitative (retrospective case-study) - Content analysis of parliamentary sessions’ transcripts, policy documents, gray literature and published papers and articles including 472 policy documents - 6 interviewees (both exploratory and reflective interviews) and 15 interviewees (reflective interviews) with key informants that were involved in or were affected by the development and implementation of the policy
Doshmangir 2015 [21]	Article, board of trustees policy	• Exploring the perceptions and views of expert stakeholders as to why the BT policy did not achieve its perceived objectives • Providing an in-depth understanding of the implementation of BT policy in the Iranian health system	Health system experts including policy-makers, healthcare managers, members of selected hospitals’ boards of trustees, and health care providers	• Qualitative - 47 semi-structured face-to-face interviews and two focus group discussions (involving 8 and 10 participants, respectively) and analysis of a comprehensive set of relevant documents
Masoudi Asl 2015 [22]	Article, board of trustees policy	• Comparing performance indicators of two gynecology hospitals with different organizational governance (budgetary/BT)	2 gynecology hospitals (Tabriz)	• Quantitative (cross-sectional and correlation study) - Five variables of hospital performance (quality management, safety, medical equipment management, and patients and staff satisfaction) was collected during the years 2011 to 2013 using standard lists and questionnaires
Joudaki 2015[23]	Report, corporatization policy	• Comparing corporate hospitals and budgetary hospitals in terms of performance indicators	3 corporate hospitals and 3 budgetary hospitals, all affiliated with the SSO	• Quantitative (cross-sectional retrospective study) - Data was collected using hospital information system (HIS) and statistical reports sent by corporate hospitals and the Province’s Health Administration
Mehrolhassani 2017 [24]	Article, board of trustees policy	• Determining the allocation of financial resources in one BT hospital	1 teaching hospital (Kerman)	• Qualitative study-case study - Review of documents, two in-depth interviews and five focus group discussions with eleven experts). Participants were members of the board of trustees and representatives of the financial department (
Zahmatkesh 2017 [25]	Thesis, hospital autonomization policy and board of trustees policy	• Exploring the extent to which hospital middle managers can exercise autonomy in England and Iran • Explaining the impact of public management reforms on middle managers and their response to these reforms	2 first-wave applicants for FT status in England, and 2 public hospitals in Iran (Isfahan) that have become BTs	• Qualitative (Comparative case study) - Face-to-face semi-structured interviews with 45 middle managers - Observational fieldwork - Documentary analysis
Jafari 2018 [26]	Article, board of trustees policy	• Identifying the barriers in implementing the board of trustees policy	All Iranian medical sciences universities and hospitals, including 56 hospitals that implemented the BT policy	• Qualitative - The survey forms were officially mailed to participants. Then, it was followed up by phone calls for further description. The survey form had 2 main questions describing the barriers and proposing the solution
Doshmangir 2019 [7]	Article, hospitals autonomy policy and board of trustees’ policy	• Analytical, sequential and chronological exploration of key historical events, achievements and challenges within the Iranian health system during the past four decades	parliament, the MOHME, the Iranian Health Insurance Organization, the Social Security Organization, the Iranian Academy of Medical Sciences, newspaper and journal articles and social media	• Qualitative document analysis • Comprehensive document and publications review(Data were collected from various sources mentioned in setting)
Mohammadi 2019 [27]	Article, corporatization policy	• Identifying and explaining the role and importance of factors affecting hospital holding administration (Milad-e-Salamat Institute) in the SSO	Healthcare Holding of the SSO (Milad-e-Salamat Institute)	• Mixed methods (qualitative and quantitative study) - Literature review and an interview with 15 key informants to identify key factors affecting healthcare holding administration to develop a questionnaire • Questionnaire completed by 405 staff, line managers, and experts working in the SSO (health management(
Azami_ Aghdash 2019 [28]	Research project, corporatization policy	• Comparing public, private, corporate, and budgetary hospitals in terms of performance indicators • Qualitative evaluation of the current structure of hospital governance • Providing a model for reforming the structure of the hospitals affiliated with the SSO	2 corporate hospitals with 5 budgetary hospitals, all affiliated with the SSO	• Mix method (qualitative and quantitative study). Data collected using: - Input, throughput, and output indicators collected by data collection sheet, HIS, facility-level statistical reports, and medical records - Likert-based leadership style questionnaire - Inpatient and outpatient satisfaction survey - Hospital Survey on Patient Safety Culture - Semi-structured face-to-face interviews with key informants

### Hospital autonomization policy

Organizational hospital reforms in Iran began in 1995 with the issuance of guidelines by the MOHME for new hospital administration called the fee-for-service plan. According to these guidelines, which became known as the hospital autonomization plan, public hospitals were allowed to generate revenues from cash payments and insurance premiums, thus reducing their dependence on government budgets. Despite the attempts by stakeholders to continue the plan, especially by the Planning and Budgeting Organization, it faced opposition by both the MOHME and the Parliament and was terminated in 1996 following the approval of a plan for financing the salaries of hospital staff from the government budget.

Jafari et al. [11, 12] conducted two studies to determine the key organizational dimensions that influence the autonomy of public hospitals using World Bank’s organizational reform model and assessed the degree of autonomy granted to the hospitals based on these dimensions. In the first step, nine themes representing key organizational elements were identified, including decision rights in “strategic”, “human resources”, “financial” and “physical resources” management, “product” and “procurement” market exposure, “residual claimant”, “governance arrangements and accountability mechanisms”, and “social functions”. In the next step, they assessed the degree of autonomy granted to the hospitals on the basis of these nine themes through interviews with 32 senior managers. The results indicated very limited decision right in “strategic”, “human resources” and “physical resources” management. Hospitals were allowed to generate revenue (fee-for-service) but were not the sole residual claimants. In addition, hospitals were exposed to the product market but were limited in the procurement market (payment ceiling). Hierarchical and financial accountability were the main accountability mechanisms. Several insurance programs and the governmental budget were used to protect poor people. The results of these studies indicate that hospital autonomization was unbalanced and inconsistent. More decision rights in “strategic” and “human resources” management and higher procurement market exposure should be granted. Also, the hospital should be the sole residual claimant. The government needs a regulatory and accountability mechanism to ensure high hospital performance outcomes and balance revenue generation and social objectives.

Another study by Doshmangir et al. [20] describes and assesses the development and implementation of the hospital autonomization policy to understand the intentions and motivations of policymakers, general outcomes of the policy, and the reasons behind the perceived failure to achieve its intended objectives. The findings indicate that stakeholders of the policy had different and conflicting objectives, which resulted in an unsatisfactory implementation process. This policy led to long-lasting and often negative changes in the hospital sector and the entire Iranian health system. Hospital autonomization appeared to be an ill-advised policy to remedy the inefficiency problems in low socioeconomic areas of the country. The idea that hospital autonomization would necessarily result in a better health system may be a false assumption, as its success relies on many contextual, structural, and policy implementation factors.

Two quantitative studies have studied the effect of autonomy policy on hospital performance. Amiri [10] examined the effect of autonomy on the performance indicators of 5 teaching hospitals before and after the implementation of the autonomy policy and its results indicated the policy failed to meet the expected objectives to improve service quality and efficiency. Ahmadi [17] investigated the effect of autonomy policy on inpatient satisfaction as one indicator of service quality before and after implementation of autonomy policy using questionnaires based on the SERV-QUAL model. This study showed that the implementation of autonomy policy had a significant effect on patient satisfaction.

Another quantitative study by Meshki [19] examined the relationship between autonomy policy and performance from the perspective of 55 Executive and Accounting Managers and Clinical staff in 5 hospitals affiliated to Gilan University of Medical Sciences. The results of the study showed that there was a significant relationship between autonomy policy and financial performance, but poor implementation of the autonomy policy led to failure to achieve its goals.

### Board of trustees hospital policy

The first wave of organizational hospital reforms began with the hospital autonomization policy and entered a new phase as a result of the failure to achieve its objectives and in line with the Fourth and Fifth National Development Plans. The new phase involved the development of board of trustees (BT) hospitals. According to Article 88 of the National Fourth Development Plan (2005–2009), the MOHME is required to take measures to increase productivity and make optimal use of national healthcare resources. Paragraphs B and C of this article highlight the need for customer focus in healthcare centers, reforms of management processes and structures, and having hospitals governed by board of trustees with established tariffs [29].

Paragraph D of Article 32 of the Fifth Development Plan also emphasizes the establishment of board of trustees in teaching hospitals. This initiative is supported in annual national budgets. According to the guidelines on administration of BT hospitals, the main objectives of this policy are continuous improvement of quality of care, enhancement of clinical care, increasing productivity, timely provision of services, and increasing patient satisfaction as well as establishment of employee performance management, operational budgeting, outsourcing, maintenance management, comprehensive hospital information and management systems. This policy was implemented in 2005 and is still ongoing [30].

The main difference between these hospitals and the autonomous hospitals of 1995 is that BT hospitals are not subject to financial and trade regulations and are administered by a board of trustees. The boards of trustees consists of the following members: the chancellor of the medical university, the hospital president (secretary of the board), a health management expert, two faculty members, a representative from charitable organizations, and a mayor or a member of the municipality [21].

Ferdosi et al. [16] conducted a study to identify the stakeholders and the accountability system of two BT hospitals in Isfahan. The results of interviews with eight senior managers showed that despite the emphasis on the presence of representatives from civil societies such as the municipality in the board of trustees, their presence was limited and there was virtually no significant change in the accountability system.

Markazi Moghaddam et al. [18] studied the obstacles and barriers to the implementation of the BT hospitals policy. Nine obstacles were identified, including “board structure and composition”, “delays in the announcement of BT hospitals’ tariffs by the MOHME”, “Lack of commitment by insurance organizations and the MOHME to agreements regarding the financing of BT hospitals”, “Poor follow-up on BT policy implementation”, “irregular board meetings”, “absence of external overseers”, “shortage of full-time physicians”, “lack of management stability”, and “insurance organizations’ delayed payments”. The results suggest that unsustainable financing of this policy and the resulting financial burden on the MOHME, and insurance organizations are the most important barriers to its success, as these organizations have been unable to fulfill what they had agreed upon obligations. Changes in insurance organizations following a change in government and renegotiating on agreements by new officials is another factor that could contribute to the failure of the policy. Delayed announcement of tariffs for BT hospitals by the MOHME is likely due to the government’s concern about financing its costs. The appointment of the university chancellor as the chair of the board indicates the reluctance of the government to grant greater autonomy to BT hospitals and signals its attempts to control them through various mechanisms, thus hindering the implementation of this policy. Moreover, the results of this study showed that there were also some contextual factors that challenge the success of the BT hospital policy, including dual practice that prevents full-time employment of physicians in these hospitals.

Doshmangir et al. [21] explored the views of expert stakeholders on the reasons for the perceived failure of the BT hospital policy to achieve its objectives. The results showed that the attempts by MOHME to maintain its authority, the inability of hospitals to use the granted autonomy, unsustainable financing, poor interaction with stakeholders, not considering the context of the policy, and absence of evidence-based policymaking were the most important factors contributing to the failure of the policy. The results also suggested that the full implementation of the policy, especially some of its key aspects such as financing, could contribute to the achievement of its expected goals.

The results of Mehrolhassani et al. [24] on resource allocation in a BT hospital showed that most decisions are made by the chair of the board of trustees and the executive director and that there is no systematic and transparent process for decision making about various issues and priorities. Members of the board of trustees do not play a significant role in making decisions and policies or setting strategies, but rather have a more prominent monitoring role and only report mainly concerning the performance outcomes of the hospital. There are no clear criteria for the allocation of resources, and payments are mainly made based on the hospital’s needs. Moreover, political pressures from higher authorities also have a significant effect on resource allocation. For instance, interest groups could pressure the hospital to purchase laboratory equipment through the department of treatment. In addition, the results of this study indicated that financial issues and challenges of the hospital affect decision making and may lead to the transfer of the financial burden to the patients.

Zahmatkesh [25] examined the degree of autonomy granted to hospital middle managers in England and Iran. She found that their autonomy is constrained in both countries. In England, middle managers have sufficient financial and human resources but have to adhere to government policy and targets. Iranian middle managers are not as constrained by government policy and targets but do not have the financial and human resources necessary to exercise their autonomy. In both countries, the central government control is a major factor and affects the autonomy of hospital middle managers.

In a qualitative study, Jafari et al. [26] identified the barriers to implementing the BT hospital policy. These barriers are classified into 9 categories, including problems related to implementation regulation, financial problems in policy implementation, problems related to faculty members, the ambiguity of implementation regulations, problems related to the BTs, authority level, hospital structure, the quality and quantity of hospital human resources, and the fee-for-service system. This study finds that “implementation regulations” and “financial problems” constitute over 50% of the barriers. Partial implementation of the policy and insufficient budget are identified as the most critical factors hindering the implementation of the BT hospital policy.

Doshmangir et al. [7] conducted a comprehensive document analysis and publications review to describe and interpret the major health policy initiatives including the trend towards organizational reforms in public hospitals during the past four decades in Iran. In this study, the process of organizational reforms in hospitals is briefly described.

Four studies [13, 14, 15, 22] have been carried out to examine the effect of the BT hospital policy on hospital performance outcomes. Sajadi et al. [13] conducted a quasi-experimental retrospective case study to explore the effect of this policy on hospital efficiency three and a half years before and after implementation. Bed occupancy rate (BOR), average length of stay (ALOS), and hospital income were selected as measures of hospital efficiency, and data were analyzed using interrupted time series analysis. The results showed that the BT hospital policy did not increase hospital efficiency. Gholipour et al. [15] carried out a retrospective longitudinal study to compare performance indicators (BOR, ALOS, and BTR) of two gynecology hospitals with different forms of organizational governance (budgetary/BT hospitals). Using the Pabon Lasso model, the results indicated better performance in the BT hospital compared to the budgetary hospital. Masoudi Asl et al. [22] conducted a cross-sectional study to compare performance indicators (quality management, safety, medical equipment management, and patients and staff satisfaction) in the same settings as the previous study. Variables were weighted through hierarchical analysis (AHP) and were analyzed in SPSS 17 and Expert Choice. Among the five variables, safety had the highest weight and medical equipment management had the lowest weight. On a scale of 0 to 100, the performance score of the BT hospital was 33.08 and the score of the budgetary hospital was 29.52. Thus, no statistically significant association was found between organizational structure and performance. Finally, Manavi et al. [14] investigated the financial impact of implementing the BT hospital policy. The results showed that in 2011 and before the implementation of the policy, public hospitals paid about 7026 billion rials for physicians’ salaries and 8140 billion rials for hoteling costs, with the implementation of the policy increasing these costs by 15,669 and 12,212 billion rials respectively. Therefore, it was estimated that the full implementation of the BT hospital policy would require an additional 28,000 billion rials that should be somehow financed by the MOHME and insurance organizations.

### Independent hospital policy

In April 2018, the MOHME issued the independent hospital administration guidelines, a modified version of the hospital autonomization plan that sought to address its problems. These guidelines were drafted by the Management Structure and Technology Committee of the MOHME in line with article 2 of the Financial and Transactional Bylaw of Universities and Faculties of Medical Sciences. According to the MOHME, the goals of this plan were continuous improvement in quality of care, higher productivity, timely provision of services, and increased patient satisfaction while limiting government control. One of the most commonly cited drawbacks of the hospital autonomization plan was the unbalanced attention given to its different dimensions. The guidelines for independent hospital administration address all the dimensions of autonomization in a balanced manner. Once these guidelines were communicated in 2018, 60 hospitals announced their readiness to participate in this process based on a predefined set of conditions and criteria. Of these, 35 hospitals, including all the hospitals affiliated with Iran University of Medical Sciences, were selected as targets for policy implementation. Later, however, the resignation of the Minister of Health and subsequent political changes (including changes in the advocates of the policy) coupled with adverse macroeconomic conditions resulted in the termination of this initiative [31, 32].

### Hospital corporatization policy in Iran

Among Upstream Documents, hospital corporatization has been mentioned in paragraph C of Article 88 of the Fourth Development Plan [29]. Unlike other organizational reforms that have been implemented in hospitals affiliated with the MOHME, the hospital corporatization policy is spearheaded by the SSO. Similar to the MOHME, the SSO has implemented various hospital organizational reforms in response to internal and external challenges. One of these attempts was to have SSO-affiliated hospitals governed by a board of trustees. This policy was proposed and approved in late 2000. According to this policy, a board of trustees would be nominated by the SSO with relative authority to manage the hospitals. The 2002 budget agreement anticipated the implementation of this policy in six hospitals, and the number of these hospitals increased in 2004 and 2005. Despite the announcement of the members of the boards of trustees, practically none of the hospitals seriously entered the implementation phase of the policy.

The SSO initiated the first instance of hospital corporatization by transferring the control of Sadr Hospital to Hekmat Medical Group in 1993. The establishment of Milad Hospital as the largest hospital affiliated with the SSO and running it as a commercial institution with an independent board of directors was another attempt to improve hospital governance. The hospital corporatization policy involved the transfer of hospitals to independent corporations that were established for that purpose. This policy was first reflected in the provisions of the SSO’s 2006 budget and later emphasized in its 2007 and 2008 budgets. However, only Alborz Hospital was corporatized and five other hospitals failed to implement the policy. The provisions of the 2010 budget called for the termination of the policy except for Alborz Hospital. In the same year, the BT hospital policy was once again placed on the agenda and was approved in the 1388th meeting of SSO’s board of directors. Subsequently, a draft of bylaws for running hospitals by board of trustees was prepared by the Department of Treatment of SSO, but it was not well received by other departments. Due to widespread criticism, the Department of Economy and Planning proposed hospital corporatization policy [23].

Between 2013 and 2017, hospital corporatization was more strongly promoted. During this period, an attempt was made to run newly established hospitals as corporations. This trend continued with the establishment of the 100-bed Abolfazl Abbas hospital in Birjand (Milad 3) in 2015, the 36-bed Zagros Martyrs Hospital in Ilam (Milad 4) and the 100-bed Amir Kabir Hospital in Ahwaz (Milad 5) in 2017, and Amir Al- Momenin Hospital in Khoy (Milad 6) in 2018. Based on the 2016 resolution of SSO’s board of trustees and the 2017 resolution of its board of directors for establishing a Healthcare Holding, Milad-e-Salamat Institute was registered in 2017 and was authorized to administer the ordinary and extraordinary general assemblies of the corporatized hospitals [33].

In a study by Mohammadi et al. [27], 5 factors and 28 components that affect SSO’s Healthcare Holding were identified, including strategic planning (4 components), decision rights (6 components), financing (6 components), monitoring and evaluation (5 components), and accountability (7 components). Financing and monitoring and evaluation were found to be the most important factors.

In a report published by the Health Economics and Planning Group [23], the performance of 3 corporate and 69 non-corporate hospitals affiliated with the SSO were compared in terms of efficiency, quality, access, and accountability. Corporate hospitals had better performance in terms of efficiency (BOR, BTR, ALOS, and bed turnover interval). The revenue-to-cost ratio was greater than 1 in all corporate hospitals, while in non-corporate hospitals, only 58% of the costs were on average covered by the revenues. No significant difference was observed between corporate and non-corporate hospitals in patient satisfaction as a measure of quality of care. In terms of accountability, the results showed that the introduction of balance sheets and profit and loss statements increased financial accountability in corporate firms. A review of corporate hospitals’ invoices and records by the Office of Medical Records showed that corporatization had indeed improved performance accountability; however, the severed ties between these hospitals and the Department of Treatment reduced accountability in certain treatment and management areas.

In a research report by Azami-Aghdash [28], the performance outcomes of 5 budgetary hospitals and 2 corporate hospitals (Alborz and Sadr Hospitals) were compared based on 49 performance indicators. In the quantitative phase, the results showed better performance by budgetary hospitals in some indicators and by corporate hospitals in other indicators, and overall, the differences were not statistically significant. In the qualitative phase, 26 weaknesses and 12 strengths were identified for budgetary hospitals as the dominant governance structure. This study recommended maintaining the current structure while suggesting various changes and reforms to improve it. Some of these reforms included changes in payment and human resource management systems and in management structure by creating a board called the “High Decision Making Board” that would consist of 8 administrative and clinical staff members along with the director of SSO’s Department of Treatment.

## Discussion

### Summary of evidence

Table 4 provides a summary of organizational hospital reforms in Iran based on Preker and Harding’s spectrum along with the start and end dates of each policy, hospitals included in each policy, related studies, and key findings. Studies have shown that the hospital autonomization policy has failed to achieve its main objectives, i.e. to improve efficiency and quality. Unbalanced and paradoxical autonomization, limited decision rights regarding human, physical, and financial resources as well as strategic management, maintaining the bureaucratic structure of the hospitals instead of participatory structures such as board of directors and board of trustees, unsustainable funding, lack of alignment between autonomization reforms and financing and payment reforms, lack of evidence-based policymaking and dearth of research on hospital autonomization years after its implementation, ideological focus and the disregard for the context of the policy, and poor governance arrangements are the most important reasons for the failure of the hospital autonomization policy in Iran. In terms of financing, the main barriers to the success of this policy were unrealistic tariffs in the public sector and higher tariffs in the private sector, the prevalence of unethical activities such as under-the-table payments to health care providers and the subsequent hand-picking of patients, linear instead of global budget allocation, the inefficient fee-for-service payment system, and the fact that some hospitals were not the sole residual claimant, while others were subsidized by the MOHME [10, 11, 12, 17, 19, 20].

**Table 4:** Main characteristics and key findings of hospital organizational reforms in Iran

Preker Spectrum	Autonomization			Corporatization
	Hospital autonomization policy	Independent hospital policy	BT hospital policy	Hospital corporatization policy
**Start date**	1994	2017	2005	1993
**End date**	2005	cont.	cont.	cont.
**Ownership**	MOHME	MOHME	MOHME	ISSO
**Included hospitals**	41	35	56	7
**Included studies**	7	0	10	3
**Key findings**	•The autonomy granted to the hospitals was unbalanced and paradoxical. •More decision rights should be granted for management of strategic, human, physical, and financial resources. •The hierarchical bureaucratic structure should be transformed into a modern participatory structure. •Governance and regulatory mechanisms were ineffective. •Stakeholders with different and conflicting objectives led to the inefficiency of the policy during the implementation phase. •Focusing on ideology and ignoring the context of policy led to its failure. •Parallel reforms should be made in financing and payment methods) such as setting tariffs based on scientific principles and prospective payment systems) •Disparities in tariffs between public and private sectors reduced the ability of autonomous hospitals to compete in the healthcare market. •Some hospitals were not the sole residual claimant and some hospitals were subsidized contrary to the principles of autonomization. •Little attention was given to evidence and there was a dearth of research on the hospital autonomization policy years into its implementation.	•There is no evidence so far	•Different dimensions of the policy were not implemented in a balanced manner. •In theory, with the modern structure of the board of trustees, autonomy was granted to hospitals, but in practice, these hospitals are still controlled by the central government. •Unsustainable financing and inefficient payment system hindered the successful implementation of the policy. •Due to financial burden, the policy was not supported by insurance organizations. •Poor interaction with key stakeholders, especially insurance organizations, resulted in unsustainable financing. •Evidence on policy evaluation, especially the number of studies, has increased, but other aspects of policy such as the appropriate structure and composition of the board of trustees, regulatory arrangements, performance reporting methods and hospital accountability have received less attention. •The structure of the board of trustees, including the number of board members and board composition, were inappropriate and board sessions were not held regularly. •Inefficient governance and regulatory arrangements such as ambiguity in implementation laws s and unclear accountability mechanisms •Decision rights regarding human resource management were limited. •The results of quantitative studies do not indicate better performance of board of trustees hospitals than budgetary hospitals	•Scientific evidence on the efficiency and effectiveness of the policy is lacking even in 2020, i.e. 22 years after its implementation (1993). •The results of quantitative studies are not generalizable due to a small sample size or unreliable methodology. •Financing, monitoring and evaluation, and changes in payment and human resource management systems are considered the most important factors that affect SSO’s Healthcare Holding •poor governance mechanisms, weak internal controls, low commitment to corporate governance, and poor regulatory arrangements are among the possible reasons for the failure of this policy, but further research is needed

Although the implementation of the BT hospital policy was a step forward, it was hindered by the same problems that led to the failure of the hospital autonomization policy. The BT hospital policy was also implemented in an unbalanced manner and some of its key aspects were overlooked [21, 26]. This policy transformed the bureaucratic structure of hospitals into the more modern and participatory structure of the board of trustees. The hospitals were autonomous in theory, but various studies showed that this autonomy was limited by a variety of factors, including the appointment of the university chancellor as the chair of the board and the key decision making authority [18], the insignificant role of the other board members, the lack of a specific process for decision making, and the strong presence and political pressure of lobbyists and interest groups [24], absence of civil societies such as municipalities and governorates in board sessions [16], and centrally imposed constraints on human resource management by hospitals [21], and the government still maintains its authority in BT hospitals.

Unsustainable financing and inefficient payment systems have been cited in most studies of BT hospitals as critical factors in the failure of the policy. Lack of commitment on the part of insurance organizations to cover the agreed-upon tariffs due to the heavy financial burden of BT hospitals, delayed payments by insurance organizations [18, 21], the delayed announcement of new tariffs by the MOHME, and the resulting financial burden [14, 18], and unrealistic tariffs [13] are some of the issues highlighted in various studies. Setting tariffs should be based on scientific principles, evidence, and actual costs to increase motivation and competitiveness in the healthcare market and preventing destructive phenomena such as dual practice of physicians, referring patients to private hospitals, and wide income gap between private and public sector physicians. Revision of the hospital tariff system to increase hospitals’ revenues and financial independence, and shifting from retrospective to prospective and mixed payment systems are among the other parallel reforms that can be done to improve financing and payment system at the macro level.

Poor interaction with key stakeholders such as insurance organizations, the MOHME and the High Insurance Council are other factors that contribute to unsustainable funding and hinder the implementation of the policy. In one study, the interviewees attributed the failure of the BT hospital policy to the lack of support from insurance organizations. Therefore, good interaction between purchasers, providers, and governing bodies has been considered a critical factor in the success of this policy [21].

Lack of evidence-based policymaking and the disregard for the context of the policy [20], the inappropriate structure of the board of trustees, including the number of board members, board composition, and irregular board sessions [18], inefficient governance and regulatory arrangements, including vague implementation laws, non-compliance, and poor accountability structures [18, 21, 26], and quality and quantity of human resources [26] are other reasons for the failure of BT hospitals to achieve their goals. Organizational reform increases the authority of hospital managers at various levels, and based on the concept of Tom Busert decision-making space [34], managers can use these granted authorities to innovate and improve hospital efficiency if they have the necessary qualifications and competencies, while hospital managers did not have such characteristics. The results of quantitative studies, though not generalizable in terms of methodology and number of samples, have not been suggestive of the success of BT hospitals compared to budgetary hospitals.

Hospital corporatization is the most important reform in hospitals affiliated with the SSO. The key point in hospital corporatization reforms is the lack of scientific evidence regarding the efficiency and effectiveness of the policy years after its implementation. The low number of hospitals examined in one study and the unreliable methods used to compare efficiency in another study render their results insufficient for decision making. In another study, SSO’s Healthcare Holding, which is the ownership entity that coordinates corporate hospitals, was studied and corporate hospitals were not directly examined. Studies conducted on this policy have mentioned financing, monitoring and evaluation, and changes in payment and human resource management systems as the most important factors affecting the Healthcare Holding governance and corporate hospitals. Poor governance mechanisms, weak internal controls, low commitment to corporate governance, and poor regulatory arrangements are among the possible reasons for the failure of this policy, but further research is needed.

### Knowledge gaps in the literature on organizational hospital reforms

A review of the literature on organizational hospital reforms reveals that, despite many years being passed after their implementation, no studies were conducted on the hospital autonomization policy (only two studies 13 years after its implementation) and the hospital corporatization policy (only one study 22 years after its implementation), which would have helped to draw lessons from and improve these policies. It is necessary to conduct research in tandem with the implementation of reforms and use evidence to improve policies. The limited number of studies in this area have focused on two or several hospitals in a short period of time and their results are not generalizable. Most studies have used a few indicators for comparison, and one study that uses a variety of indicators only examines 2 out of 7 corporate hospitals. Using reliable analytical techniques such as data envelopment analysis for measuring the relative efficiency of a set of decision making units (DMUs) with multiple inputs and multiple outputs and logistic regression for examining autonomization historical data allows for a more accurate and reliable investigation of policy outcomes.

Qualitative studies have mainly focused on the reasons for the failure of a given policy or barriers to its effective implementation, and, to our knowledge, there are no studies or reports examining other components of these policies such as the structure, size, and composition of the board, regulatory arrangements, accountability mechanisms, and performance reporting. Monitoring and evaluation of policies to draw lessons from their outcomes and improve them have been largely overlooked in Iran.

## Conclusions

Various studies have shown that organizational hospital reforms in Iran have not been implemented in a balanced and proper manner. The bureaucratic structure of hospitals was changed to participatory, but in practice, these hospitals are still controlled by the government in various ways instead of indirect control such as regulatory arrangements, budget management, procurement, auditing, and performance evaluation. In most studies, unsustainable financing and inefficient payment systems have been cited as the most important reasons for the failure of these policies.

As shown by Preker and Harding [1], organizational reforms must take place parallel to other reforms, especially those related to financing and payment systems. Hence, successful reforms in Iran require evidence-based consideration of the external policy environment of the hospitals, including regulatory and funding arrangements, and the market environment, as changes in these elements affect hospital autonomy and their ability to contribute to the successful implementation of reforms. Studies conducted in Iran support this hypothesis. Most of these studies have shown that unsustainable financing is the main reason for the failure of various hospital reforms, which is itself the result of problems related to setting tariffs, inefficient payment systems, absence of strategic purchasing, and strong conflicts of interest among policymaking institutions.

In addition, some studies have shown that poor interaction with stakeholders, failure to consider the context of the health system, and lack of evidence-based policymaking are critical factors that hinder the success of organizational hospital reforms in Iran. Successful implementation of a policy in one country does not by itself guarantee its success in another country. Contextual factors and the structure of the health system, its content, and stakeholders must be taken into account in the development, implementation, and evaluation of a policy, and the policymaking process must be evidence-based so that policies are started on the right track. Dearth of methodological and timely studies on the impact of policies and achievement of their intended goals was a major problem that specifically arose in the area of hospital autonomization and corporatization policies and must be addressed by policymakers. These studies can draw lessons from the past and help improve future policies.

## Data Availability

The datasets used and/or analysed during the current study are available from the corresponding author on reasonable request.

## Abbreviations

BT: Board of Trustee
MOHME: Ministry of Health and Medical Education
SSO: Social Security Organization
BOR: Bed Occupancy Rate
ALOS: Average Length of Stay
BTR: Bed Turnover Rate
AHP: Hierarchical Analysis
DMUs: Decision Making Units
ISSO: Iranian Social Security Organization
